# Ventilation

**Published:** 1861-07

**Authors:** 


### VENTILATION.

This is a subject which should be understood by every human being, not only that man may apply it to himself, but also to the domestic animals, for their well-being is in a sense very intimately connected with our own; hence duty and humanity demand our attention to the subject; and if for the brutes that perish, much more for our servants, and, above all our children. In reference to the general subject, a contemporary remarks:

"If our people only knew how many thousands of lives they are annually sacrificing, how many hundreds of thousands are now suffering from fevers and other maladies which have their origin in the inhaling of noxious air, the excitement and alarm on this subject would be unprecedented. They are poisoning themselves by wholesale, and two-thirds of them have no suspicion of the fact.

"Our dwellings are often charnel houses. The very first necessity of every living human being—pure air to breathe—is rarely regarded in their construction. The air actually inhaled steals in at crevices and crannies, felon-like, because it cannot be shut out. Only the defects of our Architecture prevent our dying of a vitiated, poisoned, mephitic atmosphere, from which the vital element has long been exhausted. Most men, including architects, would seem ignorant of the fact that the atmosphere is a combination of different gases, only one of which is wholesome and life-giving, and that this is consumed in the lungs upon inhalation, leaving the residue to be expelled as a poison. The church, lecture-room, or other structure which is filled, or even half filled, with human beings, and its doors and windows closed, while no express provision has been made for its ventilation, very soon becomes a slaughter-pen, in which no rational being should tarry another minute. Few churches or other public edifices are sufficiently ventilated, while a large majority of them are utterly unworthy of toleration, and ought to be closed by the public authorities until they shall have been rendered fit for their contemplated use, and no longer nurseries of disease and ante-chambers to the tomb.

"Our manufactories are nearly all disgraceful to their owners and architects in regard to ventilation. They are often divided into rooms less than ten feet high, each thickly stowed with human beings, who breathe and work and sweat in an atmosphere overheated and filled with grease, wool or cotton waste, leather or cloth, and the poisonous refuse expelled from human lungs, which together are enough to incite a plague, and are in fact the primary cause of nearly all the fevers, dysenteries, consumptions, &c., by which so many graves are peopled. No factory should be permitted to commence operations until it shall have been inspected by some competent public officer, and certified to be thoroughly provided with ventilators—not windows, which *may*, indeed, be opened, but in a cold and stormy day very certainly will not be—but apertures for the ingress of fresh, and others for the egress of vitiated air, both out of the reach of ignorance, and defying the efforts of confirmed depravity of the senses to close them.

"Our bedrooms are generally fit only to die in. The best are those of the intelligent and affluent, which are carefully ventilated; next to these come those of the cabins and ruder farm-houses, with an inch or two of vacancy between the chimney and the roof, and with cracks on every side, through which the stars may be seen. The ceiled and plastered bedrooms, wherein too many of the middle-class are lodged, with no other apertures for the ingress or egress of air but the door and windows, are horrible. Nine-tenths of their occupants rarely open a window, unless compelled by excessive heat, and very few are careful even to leave the door ajar. To sleep in a tight six-by-ten bedroom, with no aperture admitting air, is to court the ravages of pestilence, and invoke the speedy advent of death. (See Dr. Hall's Book on Sleep.)

"Our railroad cars and steamboat berths are atrociously devoid of ventilation. A journey is taken far more comfortably and expeditiously now than it was thirty years ago, but with far greater risk and harm to health. There are probably ten thousand passenger cars now running in the United States, whereof not more than one hundred are decently supplied with fresh air. Most of these, wherein forty or fifty persons are expected to sit all day and doze all night, ought to be indicted as fit only for coffins. The men who make them, probably, know no better; but those who buy and use them have not even that poor excuse. They know that they are undermining constitutions and destroying lives; they know that ample means of arresting these frightful woes are at command; yet they will not adopt them, because they cost something. How long shall this be endured?"

